# Professor Jorge Hernández Franco, Instituto Nacional de Neurología y Neurocirugía Manuel Velasco Suárez (INNN, Mexico City, Mexico): Adapted Interview from the 12^th^ World Congress for NeuroRehabilitation (WCNR), Vienna, 2022

**DOI:** 10.25122/jml-2023-1018

**Published:** 2023-04

**Authors:** Andreea Doria Constantinescu, Alexandra Gherman

**Affiliations:** 1 RoNeuro Institute for Neurological Research and Diagnostic, Cluj-Napoca, Romania

**Interviewer: Andreea Doria Constantinescu**

**Interviewee: Professor Jorge Hernández-Franco**

Jorge Hernández-Franco received his medical doctor degree from the Universidad Autónoma de México, Mexico City, Mexico, in 1985, and a speciality in rehabilitation medicine, in 1989, and was certified in neurological rehabilitation by the Newcastle University, Newcastle upon Tyne, U.K., in 1999. Since 1991, he is the head of the Rehabilitation Ward at the National Neurology and Neurosurgery Institute MVS, Mexico City, Mexico, where he lectures on neurological rehabilitation. He has further lectured on physical therapy in neurological rehabilitation with the American British Cowdray Hospital since 1996. Professor Hernández-Franco has been a member of the editorial board of Developmental Neurorehabilitation since 2005 and currently serves as the vice president for Mexico, Central America, and the Caribbean within the World Federation for NeuroRehabilitation.

**A.D.C.:** Hello, Professor Hernández-Franco! We are here, in Vienna, for the 12^th^ World Congress for NeuroRehabilitation, organised by the World Federation for Neurorehabilitation. What is your first-hand opinion on this event so far, and have you participated in any previous editions?

**J.H.F.:** Thank you for the invitation to this interview! I have been working with the World Federation for Neurorehabilitation for the last 25 years, as part of the organizing committees, and I am very excited that it has developed in this way, that there are more members to the World Federation and the participation at the congresses is higher now.

**A.D.C.:** It seems that you have a very vast and nice experience with this Federation. What is the over-arching theme of this year’s congress, from your point of view?

**J.H.F.:** The theme is always transforming neurorehabilitation, and I think that is very interesting because this World Congress gives voice to all the people who would like to give their opinion, to share their work and this will provide for a very rich congress for Vienna and for the people who attend. Transforming neurorehabilitation reflects all the changes in technology and all the changes in research that we are trying to show in this event.

**A.D.C.:** How do you consider that extracorporeal shock wave therapy (ESWT) combined with botulinum toxin compares to other methods of reducing post-stroke muscle spasticity?

**J.H.F.:** There are adjuvant therapies that can help botulin toxin in regards to achieving a more efficient result when injecting it in neurological patients, more specifically in post-stroke patients. And the idea is to inject botulinum toxin, but the interval of injection shouldn’t be very short. You have to wait, for example, three months, at least, before developing antibodies. In-between this period, I think a piece of adjuvant equipment or adjuvant therapies like extracorporeal shock wave therapy, is very important. It can add some help, it can allow the physician to have better results in the treatment of these patients.

**A.D.C.:** When will established practical guidelines be available on standard parameters on ESWT in treating spasticity?

**J.H.F.:** This is a very good question because different protocols are being used in different countries. I was recently in São Paolo, Brazil, and the physicians there, mainly neurologists, were showing a book that was written by all of them – most of the people that were studying extracorporeal shock wave therapy, and they tried to establish these parameters of how protocols should set the treatment for extracorporeal shock wave therapy and physical therapy in spasticity. But there are different protocols all over the word, I would say, so we still need more studies and we need to have more communication between specialists who are using extracorporeal shock wave therapy.

**A.D.C.:** How does botulinum toxin compare to other treatments for post-stroke spasticity?

**J.H.F.:** Botulinum toxin is a very powerful tool that allows the physician to shape the activity of the muscles – the muscles that are active in stroke patients are called spastic muscles, and this spasticity or this over-activity, this muscular over-activity, can prevent somehow the patient from completing a movement or from having an efficient movement, a movement that has a significance to him. So, injecting botulinum toxin weakens, therapeutically speaking, the activity of the muscle that is opposing to normal movement. So, we can inject it, in a very specific way, in what is called ‘*the antagonist muscle*’ and this will allow the physician to give a better treatment to the patient who had a stroke. This calls not only for the injection of botulinum toxin, not only for the use of other agents like extracorporeal shock wave therapy, but we should always keep in mind that physical therapy and occupational therapy are very important for the treatment of these patients, that this will translate into functional recovery for the patients.

**A.D.C.:** Thank you so much for accepting our invitation for the interview and or sharing your valuable opinions!

**J.H.F.:** Thank you, thank you very much!

